# Sheehan's syndrome with pancytopenia: a case report and review of the literature

**DOI:** 10.1186/1752-1947-5-490

**Published:** 2011-10-03

**Authors:** Mnif Fatma, Elleuch Mouna, Rekik Nabila, Mnif Mouna, Charfi Nadia, Abid Mohamed

**Affiliations:** 1 Endocrinology Department, Hedi Chaker Hospital, (3029) Sfax, Tunisia

## Abstract

**Introduction:**

Sheehan's syndrome is defined by varying degrees of anterior pituitary deficiency due to postpartum ischemic necrosis of the pituitary gland after massive bleeding. It is a rare disorder in western countries and even in Tunisia. Hematologic abnormalities such as normochromic anemia have been reported in these patients. However, pancytopenia is rarely observed.

**Case presentation:**

We describe the case of a 48-year-old Tunisian woman with features of hypopituitarism. Laboratory tests showed pancytopenia that was completely reversed after adequate hormone replacement.

**Conclusion:**

Clinicians should consider the possibility of hypopituitarism as a cause of pancytopenia. This is an original case report that is of interest to hematologists, who should be aware of Sheehan's syndrome as a treatable etiology of pancytopenia for women.

## Introduction

Sheehan's syndrome is characterized by varying degrees of anterior pituitary dysfunction due to postpartum ischemic necrosis of the pituitary gland after massive bleeding. The most frequent hematologic finding is anemia. Pancytopenia is rarely observed in patients with Sheehan's syndrome. Only seven cases have been reported up to now.

## Case presentation

A 48-year-old Tunisian woman was urgently sent to our intensive care department with confusion, vomiting, abdominal pain and diarrhea. A low blood pressure was observed (95/65 mmHg). An initial laboratory test showed hyponatremia at 119 mmol/L and pancytopenia. Her leukocyte count was 3 × 10^9^/L with a neutrophil count of 1.8 × 10^9^/L. Her hemoglobin level was 7.9 g/dL with mean corpuscular volume at 87.7fL, mean corpuscular hemoglobin at 32pg and mean corpuscular hemoglobin concentration at 30 g/dL. Her platelet count was at 92 × 10^9^/L. Her serum ferritin level was normal at 7.29 ng/mL. A bone marrow biopsy revealed hypocellular marrow with hypocellular cell trails. A detailed interview revealed that she had excessive bleeding in the course of her first delivery at the age of 22 years. In addition, lactation failed and menstruation did not resume. She also complained about generalized weakness. A physical examination showed pallor and doughy skin. Her breast tissue was normal but the areolae were depigmented. She had no pubic or axillary hair. Pituitary hormone studies were compatible with a status of primary pituitary insufficiency (Table 1). In fact follicle-stimulating hormone was at 3.6 mIU/mL, luteinizing hormone was at 0.6 mIU/mL and estradiol less than 9 pg/mL. Free thyroxine was low at 1.8 pmol/L with an inadequate level of thyroid-stimulating hormone at 3.9 μU/L. Her basal serum cortisol (measured at 8 a.m.) was 20 ng/mL, cortisol (measured at 4 p.m.) was at 19.5 ng/mL and cortisol (measured at 11 p.m.) was 18.1 ng/mL. After a synacthen stimulation test, cortisol was at 36 ng/mL. Her prolactin level was 2.8 μg/L. The diagnosis of Sheehan's syndrome was established and pituitary magnetic resonance images demonstrated an empty sella. She received replacement therapy with L-thyroxine 100 μg/day and hydrocortisone 20 mg/day.

**Table 1:** Hematological anomalies in previously published cases and our patient [5]

Case	Ref	Hb g/dL	TLC × 10^3^/μL	MCV fL	MCH pg	MCHC g/dL	PLT × 10^3^/μL	Reticulocyte %
1	[6]	9	3.4	87	31	36	71	0.4

2	[7]	6.5	2.3	85	33	35	96	0.2

3	[4]	7.5	3.42	96.9	32.8	33.8	70	0.9

4	[8]	9	2.3	-	-	-	90	0.3

5	[5]	9.2	3.2	94.2	30.7	32.4	64	0.3

6	[5]	10.6	3.4	82.2	26.4	32.1	74	0.5

7	[5]	9.8	3.2	94.8	33.1	31.6	42	0.2

8	Present case	7.9	3	87.7	32	30	92	

Hb: hemoglobin; TLC: total leucocyte count; PLT: platelets; MCV: mean corpuscular volume; MCH: mean corpuscular hemoglobin; MCHC: mean corpuscular hemoglobin concentration.

Our patient was followed for six weeks after which a complete hematological recovery was noted, with a eucortisolemic and euthyroid state. In fact, the hematologic abnormality had dramatically improved. Her leukocyte count was 6.2 × 10^9^/L, her hemoglobin level was 10 g/dL and her platelet count was 219 × 10^9^/L, all within the normal range.

## Discussion

The diagnosis of Sheehan's syndrome is determined by the patient's history and physical examination, and confirmed by laboratory tests (hormone levels and hormone stimulation tests which prove anterior pituitary failure). Laboratory tests can reveal many other anomalies such as hyponatremia. This is the most common electrolyte imbalance, occurring in 33% to 69% of cases [1,2]. Cortisol deficiency, hypothyroidism and volume depletion are the main causes of hyponatremia. It also seems that Sheehan's syndrome has hematological consequences, to which little attention is paid because of their rarity. Anemia is well recognized as a feature of hypopituitarism. Gokalp *et al. *have recently reported hematological abnormalities in 65 patients with Sheehan's syndrome, 80% of whom presented with anemia, compared with 25% of controls [3]. Many hormonal deficiencies, such as hypothyroidism, adrenal insufficiency and gonadal hormonal deficiency, can explain normochromic anemia in hypopituitarism [4]. On the other hand, it can be the result of a physiologic adjustment to lower oxygen requirement, as pituitary hormones modulate the production of erythropoietin in the kidney [5]. The low erythropoietin levels found in these patients support this argument. However, within the framework of hematologic disorders, pancytopenia is rarely observed in patients affected with Sheehan's syndrome.

A literature review revealed the rarity of this disorder. The first case was reported by Ferrari *et al. *in 1975 [6]. Only seven cases have been reported up to now (Table 1 and Table 2) [5]. The mean age of these women was 41.1 years (range: 22 to 57 years). The mean time between the hemorrhagic accident and diagnosis was at 11.1 years (range: two to 27 years) [5,7,8]. Hematologic disorders were behind the discovery of Sheehan's syndrome in four cases. Hormonal investigations confirmed thyroid, adrenal and gonadal deficiency in all cases. A bone marrow biopsy was carried out for all patients showing hypocellularity and decreased hemopoiesis, erythropoiesis and granulopoiesis. Pancytopenia as a result of an anterior hormone deficiency has not been clearly investigated. It is a consequence of the loss of effect of pituitary hormones on metabolic reactions to hematopoiesis, which is related to hypopituitarism [4,5]. How the anterior pituitary insufficiency can lead to complete marrow aplasia has not been clear until now. Treatment with thyroxine and glucocorticoides led to full hematological recovery in all published cases after a eucortisolemic and euthyroid state was attained after a follow up of 20 days to four months. For our patient, hematological recovery was obtained after six weeks.

**Table 2:** Hormonal results of all reported patients [5]

	Case 1	Case 2	Case 3	Case 4	Case 5	Case 6	Case 7	Our patient
T4 μg/dL	1	0.9	0.23	0.43	1.8	1	< 1	1.8

TSH μU/mL	?	0.34	1.9	1.4	0.39	2.9	1.49	3.9

FSH IU/L	0.3	1.6	1	0.58	2.9	1.07	7.43	3.6

LH IU/L	0.8	1.1	1.6	0.18	0.74	1.07	0.75	0.6

PRL μg/L	-	-	-	-	2.8	1	1.9	2.8

Cortisol μg/dL	4	-	0.7	< 1	5.8	< 1	6.6	20

GH μg/L	0.3	0.08	-	< 0.05	< 0.25	< 0.25	0.24	-

ACTH ng/mL	-	< 1	12	< 10	-	-	-	-

ACTH: adrenocorticotropic hormone; FSH: follicle stimulating hormone; GH: growth hormone; LH: luteinizing hormone; PRL: prolactin; TSH: thyroid stimulating hormone; T4: thyroxine.

## Conclusion

Pancytopenia is a rare appearance of a hormonal abnormality; clinicians should consider the possibility of hypopituitarism as a cause of pancytopenia and indicate a series of hormonal examinations. Multiple anterior pituitary hormone deficiencies in Sheehan's syndrome can be responsible for pancytopenia. A simple replacement therapy with thyroid and cortisol hormones results in complete recovery. So, hematologists need to be aware of Sheehan's syndrome as a treatable etiology of pancytopenia in women.

## Patient's perspective

Since the postpartum I suffered from fatigue, pallor, anemia and amenorrhea. Recently I presented with vomiting and diarrhea. Biological findings showed pancytopenia and hypopituitarism. After starting hormone replacement treatment, I felt better. I prefer to remain anonymous.

## Consent

Written informed consent was obtained from the patient for publication of this manuscript and any accompanying images. A copy of the written consent is available for review by the Editor-in-Chief of this journal.

## Competing interests

The authors declare that they have no competing interests.

## Authors' contributions

MF analyzed and interpreted the patient data regarding the endocrinological disease and was a major contributor in writing the manuscript. EM analyzed and interpreted the patient data regarding the endocrinological disease and was a major contributor in writing the manuscript. RN interpreted the patient data regarding the hematological disease. MM performed the bone marrow biopsy. CN interpreted the patient data regarding the hematological disease. AM analyzed and interpreted the patient data regarding the endocrinological disease. All authors have read and approved the final manuscript.
